# Coping Strategies of Family Members of Hospitalized Psychiatric Patients

**DOI:** 10.1155/2011/392705

**Published:** 2011-05-17

**Authors:** Phyllis M. Eaton, Bertha L. Davis, Pamela V. Hammond, Esther H. Condon, Zina T. McGee

**Affiliations:** ^1^School of Nursing, Old Dominion University, 2138 Health Sciences Building, Norfolk, VA 23529, USA; ^2^School of Nursing, Hampton University, Hampton, VA 23668, USA; ^3^Sociology Department, Hampton University, Hampton, VA 23668, USA

## Abstract

This exploratory research paper investigated the coping strategies of families of hospitalized psychiatric patients and identified their positive and negative coping strategies. In this paper, the coping strategies of 45 family members were examined using a descriptive, correlational, mixed method research approach. Guided by the Neuman Systems Model and using the Family Crisis Oriented Personal Evaluation Scales and semistructured interviews, this paper found that these family members used more emotion-focused coping strategies than problem-focused coping strategies. The common coping strategies used by family members were *communicating with immediate family, acceptance of their situation, passive appraisal, avoidance, and spirituality*. The family members also utilized resources and support systems, such as their immediate families, mental health care professionals, and their churches.

## 1. Introduction

Currently, there are over 57 million Americans (about one in four adults) who have a diagnosable mental disorder [1]. Studies have indicated that families caring for individuals with mental illness suffer from increased levels of stress and find it difficult to cope with their situation [2, 3]. Families experience economic strain, isolation, burnout, and real and attributed stigma when living with a family member with mental illness [4, 5]. The direct cost (mental health services and treatment) and indirect cost (related to the disability) of mental illness are in excess of $300 billion per year [1]. Mental health care in the United States is only 6.2% of all health care expenditures, while individuals with mental illness rank only second to asthma in paid expenses for care related to their disorder [1]. Over 36 million individuals in the United States have paid $57.5 billion in 2006 for mental health services [6]. The World Health Organization estimates mental illness disorders are the leading contributor to the total burden of disability in North America and are almost double the disability burden of cardiovascular disease and cancer [1].

Coping with a mentally ill relative can be difficult, and many families are being faced with the added responsibility of transitioning their relative from inpatient psychiatric treatment to outpatient treatment [7, 8]. With about half of the mentally ill suffering from more than one mental disorder at the same time, continuation of care after hospitalization is essential for positive treatment outcomes [1, 7]. For many mentally ill individuals, Medicaid is their only source of health coverage. Several states have reduced funding to Medicaid and community mental health programs in an effort to control costs [9, 10]. Currently, Medicaid accounts for more than 50% of all public funding for mental health treatment; this is expected to rise to almost 70% by the year 2013 [11]. These proposed reductions to Medicare are estimated to be $178 billion by the year 2013 [11]. As states cut funding to Medicaid, services to community mental health centers will become more limited [9]. This lack of sufficient mental health resources results in gaps in services to individuals who are in need of continuation of care, especially after hospitalization [7, 12]. 

Currently, families find accessing community mental health treatment services to be complex and wait times for services may be weeks or even months [10]. Some families are using emergency departments for mental health treatment [13]. Approximately 67% of emergency physicians surveyed in 2004 attributed this increased use of emergency departments for mental health treatment to the recent reductions in state budgets [13]. Of these physicians, one in ten stated that there is no place in the community for these patients to receive mental health treatment. Some families see incarceration of their mentally ill relative as the only safe alternative when treatment options are unavailable. As their mentally ill family member's condition deteriorates, their behavior may become volatile and incarceration may be the only safe place available [14]. 

Currently, many states are facing economic hardship, and budget reductions to mental health services have occurred. According to one survey, states spend less on mental health services now than they did in 1955 when budgets were adjusted [15]. Since 2009, state mental health services have made reductions in services that total nearly $1.8 billion, and for many states their budget reductions total over 20% of the entire mental health budget [16]. These reductions in funding have led the state and local governments to decrease mental health treatment services. In Kansas, admissions to the state psychiatric hospitals have been frozen for the year 2010. In Mississippi, six crisis centers and four mental health facilities (including two inpatient hospitals) are in jeopardy of closing. In Illinois, funding for community-based mental health care has been decreased for over 10,000 low income children and adults [16]. These financial cuts translate into fewer available treatment options for the mentally ill.

The impact of the unavailability of services is felt by families who have been given the responsibility of caring for their mentally ill relative after psychiatric hospitalization [17]. With decreased mental health treatment options, family members have been tasked to help fill the gap in the service that exits between inpatient to outpatient treatment [7]. Families are experiencing difficulty in coping with this situation and feel unprepared to fill this gap in treatment service [18]. 

The term coping has several definitions ranging from the ability to deal with problems to, as Lazarus and Folkman defined it, a transactional process, which changes over time and within its situational contexts [19]. Pearlin and Schooler defined coping as “any response to external life strains that serves to prevent, avoid, or control emotional distress” [20, page 3]. The authors stated that the act of coping is the thing one does to avoid harm from life strains. Pearlin and Schooler also recognized that the act of coping is related to both the life strains and the state of one's inner emotional life. 

McHaffie viewed coping as an important concept of study in the field of nursing. The author stated that recognizing how well an individual copes, no matter how ill he is, will determine his psychological well-being [21]. McHaffie discussed how the traditional ideas of coping define it either as a trait or as a style. The author used the concepts of Lazarus and Folkman to define coping as a dynamic process where individuals work through situations and events [19]. The author suggested that it is the transaction between the individual and the environment which enables one to cope [21].

Hatfield and Lefley applied the concept of coping to mental illness. The authors examined how families cope with mental illness by doing what is necessary to survive through the use of adaptation and coping mechanisms [22]. These authors also viewed coping as Lazarus and Folkman described, which involves the appraisal of the experience and coping responses that evolve from the appraisal. 

M. A. McCubbin and H. I. McCubbin viewed coping as part of their resiliency model. These authors viewed adaptive coping as a family strength and defined a coping behavior “as a specific effort (covert or overt) by which an individual (or group of individuals such as the family) attempts to reduce or manage a demand on the family system” [23, page 22]. The authors also discussed how coping, in relation to multiple family demands, can be viewed as a generalized response versus a situation-specific response. 

Lazarus and Folkman supported the view that even though stress is inevitable, it is the coping that makes the difference in adaptational outcomes. The authors' theory defined coping ability as one's response to a demand and not an automatic response to the environment [19]. This theory views coping as a process that focuses on what the individual thinks and does when encountering stress and how the individual reacts as the encounter unfolds. 

According to Lazarus and Folkman, coping has two major functions: (a) regulating stressful emotions or emotion-focused coping and (b) altering the distressed person-environment relation or problem-focused coping [19]. Emotion-focused coping refers to efforts to manage the negative emotions associated with the stressful situation. The emotion-focused coping strategies help to decrease the distress. These strategies include cognitive distraction, seeking emotional support, emotional regulation and expression, selective attention, and cognitive restructuring. These authors referred to problem-focused coping as the efforts used to change the basis of stress directly. This type of coping focuses on altering the environment, changing the external pressures, or seeking resources to help make the situation less threatening. 

Problem-focused coping strategies usually are used by adult individuals in work contexts, and emotion-focused coping strategies are used in health-related contexts [19]. This shift in strategies is related to the appraisal of control in the situation. Lazarus and Folkman [19] stated that emotion-focused coping is used when events are perceived as uncontrollable, such as health situations. This type of coping is concerned with managing the emotions associated with a stressful situation. The authors stated that problem-focused coping is used when the situation is perceived as controllable, such as in work situations. This type of coping is concerned with directly managing the source of stress.

Coping strategies can be classified further as being positive or negative. Some family members use positive coping strategies to help them manage their situation, such as positive thinking and the utilization of appropriate social supports, which include family, friends, and the church [24–26]. Some families may also use negative coping strategies, such as the use of avoidance behaviors, negative thinking, and substance abuse [27, 28]. 

Communication is a coping strategy that family members use to share information about their thoughts and feelings. The social stigma of mental illness is seen as a deterrent to sharing information about their mentally ill relative outside the family. Concealment of the illness has been viewed as preferable to disclosure due to the risk of negative reaction from others [29]. Communicating with family members is considered to be an emotion-focused coping strategy and may be done through verbal or written contact [19]. Verbal communication can occur in person, via telephone or electronic devices, such as on-line video interactions. Families also communicate through written contact, such as letters, or electronically through emails, texting, or on-line social networks. Families dealing with mentally ill relatives find communicating with outside sources difficult. Many family members cope with their situation by sharing their feelings only with their immediate family, and through this, receive support and understanding [19]. 

Acceptance is another emotion-focused coping strategy that family members may utilize to cope with their mentally ill relative. Acceptance is a form of cognitive restructuring where individuals appraise their thinking reactions to situations and change negative reactions to positive, or at least neutral ones [19]. Acceptance is a strategy families may use to obtain a more accurate and beneficial view of their situation. Many family members perceive their situation as uncontrollable, and something they must deal with, and move on. Family members who use acceptance see their situation as not negative, but as a part of their everyday life. Incorporating acceptance in their life helps to reduce stress and improve their relationship with family members [30]. By cognitively accepting their difficult situation, families are able to redefine stressful events so as to make those events more manageable [19]. 

The use of cognitive distraction is also noted to be an emotion-focused coping strategy, which includes passive appraisal [19]. Cognitive distraction is the mental process of distraction. It may be something that limits attention or prevents concentration. It can also be something that distracts the mind from thinking about stressful situations and limit reactivity [19]. Passive appraisal is a form of cognitive distraction that allows the individual to accept and minimize reaction to difficult situations [19]. This coping strategy can be used to help families accept problematic issues through minimizing reactivity. Family members are able to minimize their reaction by utilizing passive appraisal activities, such as watching television, relying on luck, feeling helpless about the problem, and believing that time will solve the problem. 

Some families find it difficult to deal with the stigma associated with mental illness, and this negatively influences the coping ability of the family [31, 32]. Some family members with mentally ill relatives use avoidant strategies to cope with their situation. Avoidance is an emotion-focused coping strategy that some families use when they overestimate the threat of the illness or underestimate their own coping ability [30]. Avoidance is a behavior that limits the exposure to distressing situations. It is way of avoiding uncomfortable issues or situations. Avoidance may include removing oneself physically from a situation or refusing to discuss or even think about the issue or situation [30]. Avoidant strategies may include ignoring the family member by decreasing physical and emotional contact, such as not communicating and visiting them regularly and limiting their affection [28]. Families may also totally avoid their mentally ill relative and cut off any type of contact.

Some families use religious and spiritual support as a means for coping with caring for a mentally ill relative. The use of spirituality is seen as a positive emotion-focused coping strategy [16]. Spirituality may mean different things to different individuals, but has been seen to increase levels of well-being and decrease the level of stress in one's life [33]. It can be both intraphysical, such as through values and beliefs, and institutional, such as through church attendance and performing rituals. Spirituality includes seeking advice from a minister, attending and participating in church services, and having faith in God. Spirituality may also include prayer, privately and with a community. It has also been seen to increase levels of well-being and decrease the level of stress in one's life [33]. 

Families use a variety of coping strategies and resources to maintain healthy family functioning [30]. A vital positive coping strategy that families living with mental illness can have is utilization of social supports. Accessing social support is an adaptive problem-focused strategy [19]. Social support is described as an exchange of information by individuals, which provides emotional support, esteem support, and network support [30]. Social supports may be social networks such as churches, friends, and extended family, or they may be more formal networks such as health care or educational institutes.

As mental health treatment options decrease, families are being given the responsibility of transitioning their mentally ill relative from inpatient psychiatric treatment to outpatient treatment [7]. The coping strategies of a family member may influence the treatment outcomes of their relative positively or negatively [7]. Identification of the family member's coping strategies may aid in helping the family cope with their situation. Understanding how the family member copes with their situation is essential in providing the best outcomes for the entire family [34]. Utilizing the Neuman Systems Model of nursing as a framework [35, 36], this study investigated what coping strategies families of hospitalized psychiatric patients use and identified their positive and negative coping strategies.

## 2. Methods

### 2.1. Research Design

This exploratory research study used a descriptive, correlational, mixed method research design. This study explored the coping strategies, support systems, and resources of family members of hospitalized psychiatric patients. This design was used to determine if a relationship exists between family coping strategies and spirituality, ethnicity, gender, income, and family relation of the family members. This study used the F-COPES for the quantitative portion of the study and a semistructured interview for the qualitative portion of the study.

The Neuman Systems Model of nursing provided the framework for this study [35, 36]. The Neuman Systems Model views the client/client system as dynamic, interrelated variables that interact continuously with stressors from the environment. The client/client system can be defined as a single client, a group, a family, or a community. All client systems have five variables which are interrelated: (a) physiological, (b) psychological, (c) sociocultural, (d) developmental, and (e) spiritual. These variables refer to the (a) bodily structure and function, (b) mental processes and relationships, (c) social and cultural functions, (d) life developmental processes, and (e) spiritual beliefs that influence the client system. The five variables work together simultaneously as the client responds to the stressors of the internal and external environment [35, 36].

The basic structure of the client/client system is represented as a series of concentric rings or circles, which surround the core. The rings are divided into three different type lines: (a) the flexible line of defense, which represents the outer concentric ring (broken line) and which acts as a buffer system for the client's normal state; (b) the normal line of defense, which is the solid line that lies between the flexible line of defense and the internal lines of resistance and which represents the client's usual wellness level or steady state; (c) the lines of resistance, which represent the inner concentric circles (broken rings) and which contain internal and external resource factors which help protect the client against a stressor [35, 36].

In the Neuman Systems Model, the environment is a key concept which affects the client system. The environment is defined as all the internal and external forces affecting the client positively or negatively [35, 36]. This environment is divided into internal, external, and created environment. The internal environment includes influences internal to the boundaries of the client system. This is where intrapersonal factors or stressors (something that occurs within the person) arise. The external environment contains all influences and forces that exist outside the client system. This is where the interpersonal (something that occurs between people) and extrapersonal (something that occurs outside the person) factors or stressors arise [35, 36].

The created environment acts as an open system that exchanges energy with the internal and external environment [35, 36]. This environment is unconsciously created to help maintain the integrity of the system and is viewed as a symbol of system wholeness. This environment acts as an insulator that helps to change the response of the client to stressors. Thus, the objective of the created environment is to stimulate the health of the client.

**Figure 1:**
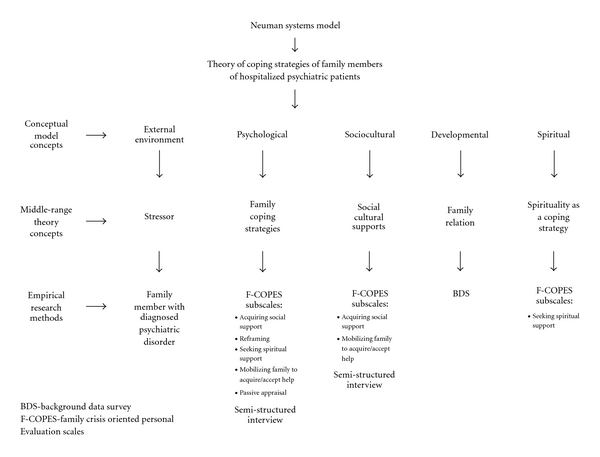
Conceptual-theoretical-empirical structure for the study of coping strategies of family members of hospitalized psychiatric patients.

The Neuman Systems Model [35] was used to guide this study and test the middle-range theory of coping strategies of family members of hospitalized psychiatric patients (see Figure 1). This conceptual model was chosen to help guide this study because it provided a holistic and system-based approach, which focused on the response of the client/client system to actual and potential environmental stressors. The client/client system represents the family member of the hospitalized psychiatric patient. 

The psychological client variable refers to the relationships and mental processes of the client system [35, 36]. Family coping strategies were used to examine the psychological functioning of the client system. The theory concept of social cultural supports represented the sociocultural client variable. The sociocultural variable addresses both cultural and social functions of the client system, which include social support and resources [35, 36]. Resources and support systems were used to identify the sociocultural function of family members of hospitalized psychiatric patients. The theory concept, family relation, represented the developmental variable. The developmental variable refers to the developmental processes in life [35, 36]. This variable identified how the developmental process of family relation impacts the coping ability of the family members of hospitalized psychiatric patients. The theory concept of spirituality as a coping strategy represented the spiritual client variable [35, 36]. The spiritual variable refers to the influence of spirituality on the client systems. In this study, the family member's coping ability was examined in relation to their use of spirituality.

The client reacts to the intrapersonal stressors that arise from the internal and created environment [35, 36]. In this study, the client is the family members of mentally ill individuals, and the intrapersonal stressors may be represented by their coping strategies and personal perception of the event. The external environment can produce interpersonal stressors, which may influence the family's use of support systems and resources.

In this exploratory research study, the following four of the client variables are represented by the appropriate theory concepts and were tested by the empirical indicators as follows: (a) psychological, which represents the coping strategies of family and was tested by the F-COPES and semistructured interview; (b) sociocultural, which represents the social and cultural supports of the family and was tested by the F-COPES and semistructured interview; (c) developmental, which represents the family's relation to the hospitalized mentally ill patient (spouse, parent, sibling, significant other) and was tested by the background data survey (BDS); (d) spiritual, which represents the use of spirituality as a coping strategy and was tested by the F-COPES. In addition, the external environmental stressor from the mental illness of the relative is also represented on the following conceptual-theoretical-empirical structure.

### 2.2. Sample and Setting

A convenience sample was used for this study. The intended sample was an adult individual who was a family member of a hospitalized psychiatric patient. A family member was considered as anyone who the mentally ill individual deemed to be family. The inclusion criteria for participation in this study were (a) an individual who was 18 years of age or older; (b) a family member of the hospitalized psychiatric patient, but who was less than 80 years of age; (c) a family member of any ethnicity, gender, and socioeconomic level; (d) a family member who was actively involved in the care of the mentally ill relative (at least weekly).

The target sample for completing the surveys was 45 participants. This sample size was chosen so that there was at least 90% power to conduct both the descriptive and regression analyses. The F-COPES score was a primary outcome of the study and was expected to have the greatest variance; it was used as the basis of the sample size calculation.

### 2.3. Data Collection Procedure

The setting for this study was a psychiatric unit of a hospital in the mid-Atlantic region of the United States. Approval to conduct this study first was obtained from the Hampton University Institutional Review Board (IRB) and then the IRB approval was obtained from the mid-Atlantic region hospital. After IRB approval was obtained, the researcher began to collect data at the hospital. Family members of the hospitalized psychiatric patient were asked to participate in this voluntary study. Family members were provided information verbally about the study upon admission and during visiting hours by staff. The principal researcher was available during visiting hours to recruit participants. This researcher was the only researcher collecting data. All participants were asked to participate in a semistructured interview in addition to completing the F-COPES survey.

Informed consent was obtained from all participants, which included a statement regarding the confidentiality of the participants. The consent provided information on the name and purpose of the study and the possible risks and benefits of the study to the participants as well as information on the possible effects of the study on the fields of mental health and nursing. A copy of the consent form was given to each participant. The consent form was kept separate from the other data collection forms.

### 2.4. Instrumentation

The BDS was developed by the researcher to obtain demographic information. This information included questions regarding gender, age, ethnicity, income, and family relation. The BDS took approximately 5 minutes to complete. The Family Crisis Oriented Personal Evaluation Scale (F-COPES) was used to measure family coping. The F-COPES is a 30-item instrument, which is used to identify problem-solving and behavioral strategies used by families in crisis or problem situations [30]. The F-COPES helped to identify coping levels of families, which can reflect the ability of the family to adapt to stressful situations. McCubbin and colleagues theorized that families with more coping behaviors will adapt easier to stressful situations than families with limited coping behaviors. Also, families who have access to a larger selection of resources tend to manage more effectively in stressful situations [30]. They referred to family resources as predictors of how the family adapts to stressful events. Family resources are indentified as the family's (a) personal resources, such as finances, education, and psychological attributes; (b) social supports, such as extended family members, coworkers; and community; and (c) family system resources, such as problem solving, managerial ability, and family cohesion. 

The instrument focuses on two levels of family interaction: (a) how the family internally handles difficult situations between its members and (b) how the family externally handles the difficult situations which emerge outside its boundaries and which affect its members. The participants used a 5-point scale to complete the F-COPES. The scores ranged from 1 to 5:1 (*strongly disagree*), (2) (*moderately disagree*), (3) (*neither agree nor disagree*), (4) (*moderately agree*), (5) (*strongly agree*). 

The five subscales designed in the F-COPES include *acquiring social support*, *reframing*, *seeking spiritual support*, *mobilizing family to acquire and accept help*, and *passive appraisal* [30]. The acquiring social support subscale is a nine-item subscale that measures a family's ability to acquire support from friends, relatives, neighbors, and extended family. The reframing subscale is an eight-item subscale that assesses the family's ability to redefine stressful events to help them be manageable by the family. The seeking spiritual support subscale is a four-item subscale that examines the family's ability to acquire spiritual support. The mobilizing family to acquire and accept help subscale is a four-item subscale that measures the family's ability to seek community resources and accept help from others. The passive appraisal subscale is a four-item subscale that assesses the family's ability to accept difficult issues minimizing reactivity. 

The F-COPES subscales are calculated and then totaled together. Some of the items in the scales need to be reversed when scoring. The higher the scores, the better the problem-solving and behavioral responses found during difficult situations [30]. The F-COPES has an internal consistency reliability of  .89. This ranges from  .69 to  .83 on the various subscales. The F-COPES has a test-retest correlation over a 4-week period of  .81. This instrument is written at a sixth grade level and took approximately 10–15 minutes to complete.

The semistructured interview consisted of four open-ended questions: (a) what coping strategies do you use when caring for your loved one? (b) What support systems do you use to help you cope with your loved one? (c) What resources do you have or would like to have to help you cope with your loved one? (d) What do you think the mental health care professionals could do to help you cope with your loved one? These questions obtained the family's perception of their coping strategies, support systems, family resources, and the role of mental health care professionals in helping them cope with their mentally ill relative. The researcher, a psychiatric clinical nurse specialist with the help of another psychiatric clinical nurse specialist, developed the semistructured interview questions.

### 2.5. Data Analysis

Analyses of the demographic data obtained from the participants were summarized by descriptive statistics. The researcher specifically studied the demographic attributes of age, gender, ethnicity, income, and family relation. Continuous variables were tested for normality using the Kolmogorov-Smirnov test. Variables with approximately normal distribution were described using means with 95% confidence intervals while nonnormally distributed or discrete variables were described using medians with the 25th and 75th percentiles. 

Coping strategies that were used by family members of hospitalized psychiatric patients were determined by each subscale score of the F-COPES. An item analysis from F-COPES was completed to determine which common support systems family members of hospitalized psychiatric patients used. A correlational analysis was used to determine the relationship between spirituality of family members of hospitalized psychiatric patients and coping ability by comparing the spirituality subscale score with the total coping scale, which did not include the spirituality subscale score.

Univariate linear regression analyses between independent variables and the total F-COPES score were performed to determine the relationship between ethnicity, gender, income, family relation, and coping of family members of hospitalized psychiatric patients. Cronbach's coefficient alphas were performed to determine the reliability of the F-COPES subscales. The level of significance was set at 0.05 and SAS version 9.1 was used for all data management and analysis.

The data from the semistructured interviews were reviewed, and content analysis was performed. From this data, patterns and trends were identified and relative frequency of each unique theme was summarized. An expert in mental health nursing, a clinical nurse specialist, reviewed the data and verified the findings. Appropriate themes identified in the qualitative analysis were accompanied by quantitative results. Because both quantitative and qualitative data were collected, methods triangulation was used in the analysis of data. Both research methods were used simultaneously to measure family coping and the use of support systems, resources, and spirituality. The simultaneous triangulation for this study was performed using the data obtained from the F-COPES and semistructured interviews. The blending of the two methods occurred during the data analysis and interpretation of the findings.

## 3. Results

### 3.1. Data Procedures

Descriptive analysis was conducted of all demographic and scaled outcomes. The F-COPES subscale and total coping scores were tested for normality using the Kolmogorov-Smirnov Test. Only the acquiring social support scale and total coping score had an approximately normal distribution. Therefore, all subscale and total coping scores are described using the median with the 25th and 75th percentiles, and both normally distributed scores are described additionally with means and 95% confidence intervals. Discrete and categorical data are described with frequencies with relative frequencies. 

The quantitative data, consisting of descriptive analysis, F-COPES [29] item analysis, Chi-square analysis, Cronbach's coefficient alphas, and univariate linear regression analysis, were analyzed using SAS version 9.1. The qualitative data obtained from the semistructured interviews were reviewed, and content analysis was performed. Data obtained from the semistructured interviews and the F-COPES were analyzed together to enhance the validity of the findings.

### 3.2. Presentation of Results

In this study, 45 individuals participated, all of whom completed both the F-COPES [29] and the semistructured interview. Of these individuals, 51.1%  (*n* = 23) were women. The leading category age range of the study participants was 48–57 years old (37.8%, *n* = 17), and the prevalent family relation to the psychiatric patient was children (35.6%, *n* = 16). The ethnicity of the participants consisted of Caucasian (55.6%, *n* = 25), African American (42.22%, *n* = 19), and Hispanic or Latino (2.22%, *n* = 1). In this sample, the annual income of the participants ranged from under $10,000 per year to over $100,000 per year. The predominant annual income was $100,000 or more per year (29.7%, *n* = 11).

The F-COPES was used to quantify the coping strategies of family members of hospitalized psychiatric patients. To determine which coping strategies were used by family members, an F-COPES item analysis was performed. The participants used all items as a coping strategy, except for Item 29: sharing problems with neighbors (Mdn = 2). The strongest coping strategy used by the participants was Item 30: having faith in God (Mdn = 5). Each subscale of the F-COPES was summarized to determine which subscale of coping strategies was used by family members.

The data analysis showed that the participants used all subscales as coping strategies. Among the five subscales, seeking spiritual support was the most employed coping strategy (Mdn = 4) while acquiring social support was the least employed (Mdn = 3.11) coping strategy. The first subscale was acquiring social support, and it measures the family's ability to acquire support from friends, neighbors, and relatives. The seeking spiritual support subscale included seeking advice from a minister, attending and participating in church services, and having faith in God. Also noted was the significant use of passive appraisal as a coping strategy (Mdn = 3.75). This subscale included watching television, relying on luck, feeling helpless about the problem, and believing that time will solve the problem (see Table 1). 

The second subscale of the F-COPES is reframing. This subscale examined the family's ability to redefine stressful events so as to make those events more manageable. The coping strategies utilized most often by family members in this subscale were the acceptance of difficulties and defining the problem in a positive way. The least employed coping strategy in this subscale was believing we can handle our own problems. 

The third subscale was seeking spiritual support. This subscale examined the family's ability to acquire spiritual support. The coping strategy family members reported using the most in this subscale was having faith in God. This item was noted as the most used coping strategy of all the subscales. The least employed coping strategy in this subscale was participating in church activities. 

The fourth subscale was mobilizing family to acquire and accept help. This subscale measured the family's ability to seek out community resources and to accept help from others. The results of this subscale indicated that family members used their family doctor and counselor more often than they used seeking help from community agencies and programs.

The fifth subscale was passive appraisal. This subscale examined the family's ability to accept problematic issues minimizing reactivity. The most used coping strategy was feeling that no matter what we do to prepare, we will have difficulty handling problems. The least employed coping strategy in this subscale was believing if we wait long enough, the problem will go away.

Cronbach's coefficient alphas were reported for all subscales of this study. All subscales in this study showed good reliability, ranging from 0.76 to 0.85, with the exception of the passive appraisal subscale (0.41). In this subscale, Item 12, watching television, showed the weakest correlation to all other items in this subscale.

The semistructured interview question, what coping strategies do you use when caring for your loved one? provided qualitative information regarding which coping strategies family members of hospitalized psychiatric patients used. The most common theme noted from the participants' responses, regarding coping with their mentally ill relative, was related to spirituality. This also was noted as the most common coping strategy subscale from the quantitative data. The majority of the participants referred to the use of prayer and having faith in God as their most common coping strategy. Almost all of the participants referenced using some type of spirituality in their responses. The following common statements were made by the participants regarding how they coped with caring for their mentally ill relative:

“Pray…Pray a lot! Life is so difficult sometimes that you need as much help as you can get. Through prayer I receive strength to go on.”

Another theme that was almost as common as spirituality was acceptance. The family members discussed how they coped with their mentally ill relative by “just dealing with it” and “mov[ing] on with life.” Some of the participants articulated that the care of their mentally ill family member was their sole responsibility; there was no one else who could or who wanted to cope with the situation. The F-COPES does not assess for this attribute, but the semistructured interview questions identified this important theme. 

The participants also stated they were able to cope with caring for their mentally ill relative through the support of their immediate family. Many of the participants felt that their family was the only one who could understand what they were going through. Their responses were similar to the F-COPES data, which indicate that the participants coped by talking with their family. 

Another common theme noted in the responses of the participants regarding their ability to cope with their mentally ill relative was involvement in passive activities such as reading or watching television. These activities also were noted as common responses from the F-COPES data. Many of the participants felt these activities offered them the opportunity “not to think,” a passive activity allowed them to “relax” and “recharge.” A few of the responses from the participants regarding how they coped with their mentally ill relative were related to positive activities, such as exercising and staying optimistic. Some of the participants felt better when they were actively involved in their relative's care.

To determine which common support systems were used by family members of hospitalized psychiatric patients, each subscale of the F-COPES was summarized. Per-item average scores in a domain greater than three indicated the domain was a support system used by the participants (see Table 2).

The support system most utilized by the participants was seeking spiritual support (Mdn = 4), which consisted of church involvement, seeking ministerial advice, and faith in God. The next highest reported support system used by the participants was mobilizing family to acquire and accept help (Mdn = 3.75), which included seeking help from similar families, community agencies, the family doctor, and professional counselors. The least reported support system used by the participants was acquiring social support (Mdn = 3.11) from friends, neighbors, and relatives. 

The semistructured interview question, “what support systems do you use to help you cope with your loved one?” helped identify the support systems of family members of hospitalized psychiatric patients. The most common responses from the participants were related to family. Most of the participants responded that their immediate families were their support systems. The church was identified as another important source of support for the participants. The F-COPES data found spiritual support as the most common support system used by the participants, but the interview responses identified the family as the major source of support. The interview also identified only a few participants who saw the physician and the counselor as forms of support.

The participants' responses indicated that illness education was the most valuable resource they could have. This resource included information about signs and symptoms, relapse prevention, and medication. The second most common resource noted from the responses was the need for support groups. Most of the participants stated they never had attended one but felt it would be something that could help them cope with their relative. Another significant valuable resource for family members of hospitalized psychiatric patients was physicians and counselors. 

The semistructured interview question, “What do you think the mental health professionals could do to help you cope with your loved one?” addressed the family member's perception of the role of the mental health professional. The participants' responses indicated that the most common theme noted was information. The participants' perceived role of the mental health care professionals was to provide them with information regarding their mentally ill relative's condition, disease process, treatment, and rehabilitation. 

The relationship between ethnicity, gender, income, family relation, and coping of family members of hospitalized psychiatric patients was determined by a series of generalized linear models (GLMs). These GLMs were analyzed using the total coping score as the primary outcome. Crude effects of each demographic characteristic were determined in a univariate GLM where demographic variables were included as class variables due to the method of data collection. Individually, demographic variables did not have a significant impact on coping ability (see Table 3). The *R* ^2^ for each of the demographic variables was so low, indicating a small level of explained variation across the categories of demographic indicators. Other variables that may lead to an increase in variability are other measures of class, such as educational level and occupational status. 

The demographic profile of participants with the lowest number of total coping scores (lowest 10%) and the highest number of total coping scores (highest 10%) were examined. A higher number of those aged 48–57 years old, males, African Americans, spouses and significant others, and with incomes under $50,000 per year were among the lowest tenth percentile in total coping scores. This lowest total coping score population was compared to the demographic profile of the overall study population using a Chi-square analysis. This analysis showed that age (*P* = .97), gender (*P* = .43), ethnicity (*P* = .49), relation (*P* = .83), and annual income (*P* = .95) were not statistically significant for this group.

In analyzing the highest coping scores, it was found that a higher number of those aged 48–57 years old, females, Caucasian, children, and with incomes over $70,000 per year were among the highest tenth percentile in total coping scores. This group with the highest total scores was compared to the demographic profile of the overall study population using a Chi-square analysis. 

The analysis showed that only gender (female) was statistically significant (*P* = .04) and that age (*P* = .94), ethnicity (*P* = .25), relation (*P* = .75), and annual income (*P* = .89) were not statistically significant for this group.

The Neuman Systems Model [35, 36] guided this study and assisted in testing the theory of coping strategies of family members of hospitalized psychiatric patients. Through the model, this study was able to identify the psychological, sociocultural, developmental, and spiritual variables that affected the coping ability of family members. The F-COPES subscales and semistructured interview investigated the theory: concept of family coping strategies related to the client variable of psychological. The sociocultural client variable, which was represented by the social cultural supports theory concept, was tested by the F-COPES subscales of acquiring social support, mobilizing family to acquire/accept help, and the semistructured interview. The theory concept of family relation, which represented the developmental client variable, was tested by the BDS. The spiritual client variable, which was represented by the spirituality as a coping strategy theory concept, was represented by the F-COPES subscale of seeking spiritual support. These variables worked together simultaneously as the family members of hospitalized psychiatric patients respond to the external environmental stressor of having a family member with a diagnosed psychiatric disorder. The results of this study showed that environmental forces influenced each variable within the client system, positively or negatively.

## 4. Discussion

### 4.1. Major Findings

Coping is an emotional or behavioral response to stress [19]. It is a process which focuses on what the individual thinks and does when encountering stress. For many families, having a relative with mental illness can be a stressor [3, 22]. Coping with mental illness can be difficult for these families. Many families believe that they do not have the necessary coping strategies to help with managing the mental illness of their relative [2].

This study utilized the Neuman Systems Model to guide the investigation of copings strategies of family members of hospitalized psychiatric patients. This model allowed for the identification of the family's coping strategies where family members of hospitalized psychiatric patients were noted to use more emotion-focused coping strategies than problem-focused coping strategies. Lazarus and Folkman found emotion-focused coping is used when the situation is perceived as uncontrollable, such as with situations involving mental illness [19]. 

An emotion-focused coping strategy family members used frequently was communicating with their immediate family. Family members felt they were able cope by sharing their feelings with their immediate family, and through this, received support and understanding. Many would telephone, text, or utilize on-line social networks to contact their immediate family and discuss their day. In doing this, the family members received emotional support in dealing with their mentally ill relative. 

This study also found that the use of acceptance was a significant coping strategy for family members with mentally ill relatives. Family members felt that coping with their mentally ill relative was a situation that was perceived as uncontrollable, and something with which they must deal with, and move on. This emotion-focused coping strategy allowed the family members to carry on by cognitively accepting their difficult situation [19]. These family members coped by making the situation a part of their life, thus, not a burden. They did not spend time worrying about what could have been done, but just incorporating it into their everyday routine. 

The use of passive appraisal, a form of cognitive distraction, was also noted as a coping strategy by family members of hospitalized psychiatric patients. Activities such as watching television and reading were used by family members to help them cope with their mentally ill relative. The use of cognitive distraction is also noted to be an emotion-focused coping strategy [19]. Qualitative data and quantitative data both suggested that these cognitive distracting activities are used frequently as coping strategies for family members. Negative, maladaptive coping strategies did not significantly impact the results of this study. Only a few family members utilized negative and/or avoidant coping strategies in coping with their mentally ill relative.

The qualitative data revealed that family members felt a support group is an important resource even though most of them have not attended one. Having a relationship with the physician and counselor was also seen as an important resource by family members of hospitalized psychiatric patients. The family members stated that the physician and counselor could provide them information about their mentally ill relative and information on ways to cope with their situation. 

There was no significant relationship between coping and the socioeconomic factors. However, when examining the demographic factors of the highest coping scores, only gender (female) was found to be statistically significant. This finding may suggest that female relatives of hospitalized psychiatric patients may be able to cope better than male relatives. 

The results showed that the majority of family members of hospitalized psychiatric patients utilize some form of spirituality to help them cope with their mentally ill relative. The use of spirituality is an emotion-focused coping strategy [29]. The family members considered having faith in God, and prayer, as essential components in coping with their mentally ill relative. Almost every participant acknowledged the importance of having some type of spiritual relationship. The statistically significant data from the F-COPES was validated by the semistructured interviews, in which the use of spirituality is the most commonly used coping strategy of family members of hospitalized psychiatric patients. This finding is consistent with pervious literature where spirituality has been shown to be an effective coping strategy for families with mentally ill relatives [33, 37, 38]. 

The family members stated that they utilized a variety of support systems. The use of social supports is a problem-focused coping strategy [19]. The data revealed that the most commonly used support system noted from the F-COPES is related to spirituality, such as attending church and seeking ministerial advice. The quantitative data revealed that family members seek support from family doctors and counselors. Conversely, the qualitative analysis showed that the most commonly used support system is immediate family.

The use of spirituality is an emotion-focused coping strategy that can decrease one's level of stress and increase one's level of well-being [19]. This is evident from the statistically significant results of the family member's coping ability and spirituality correlational analysis. Findings suggested that family members of hospitalized psychiatric patients who utilize some form of spirituality may be able to cope better than those who do not use spirituality as a coping strategy. 

In the theory of coping strategies of family members of hospitalized psychiatric patients, the theory concept of family coping strategies represented the Neuman Systems Model psychological client variable. The common coping strategies identified were communicating with their immediate family members, acceptance of their situation, passive appraisal, spirituality, and avoidance. The theory concept of social cultural supports represented the sociocultural client variable. The common resources and support systems family members of hospitalized psychiatric patients used were illness education, mental health care professionals, their church, and increased finances. The findings did not show any relationship between coping and ethnicity. 

Family relation represented the developmental variable in the theory of coping strategies of family members of hospitalized psychiatric patients. The findings showed that family relation has no significant impact on the coping ability of the family member. Only gender (female) was shown to have some correlation to coping ability. The theory concept of spirituality as a coping strategy represented the spiritual client variable. This study's findings suggested that the use of spirituality may aid in the overall coping ability of the family member of the hospitalized psychiatric patient. 

These client variables are interrelated and interact simultaneously to help the family member of hospitalized psychiatric patients cope with external environmental stressors. The family member who is diagnosed with a psychiatric disorder is the stressor from the external environment. The external environment contains all the forces and influences that exist outside the client system [35, 36]. The findings from this study suggested that environmental forces positively or negatively influence the client system's ability to cope. This finding is consistent with Neuman's [35, 36] relational proposition related to person and environment concepts. In this proposition, the client system's ability to function, through input and output, is related to the intra-, inter-, and extrapersonal environment influences. The client system interacts with the environment by adjusting to it or as a system adjusting to the environment.

## 5. Conclusion

The coping strategies of family members of inpatient psychiatric patients were explored in this study. The use of emotion-focused coping strategies, such as communication with family members and cognitive distraction, were noted to be used more frequently and effectively than problem-focused coping strategies by family members of hospitalized psychiatric patients. The support of immediate family was seen as a vital coping strategy and support system when coping with their mentally ill relative. The family member's need for information is a major theme in the results of the semistructured interviews. The information is related to illness education, support groups, and the condition of their mentally ill relative. The family member's perception of the role of the mental health care professional also is related to the sharing of information. The family members felt that mental health care professionals should not only provide them with information about their mentally ill relative but also information on how to cope with their situation. Spirituality was found to be central in the family member's ability to cope with their mentally ill relative. The use of spirituality as a coping strategy may suggest a positive influence on the family member's overall coping ability. Overall, family members of inpatient psychiatric patients coped more effectively with emotion-focused coping strategies, which included communicating with family, cognitive distraction (passive appraisal), cognitive restructuring (acceptance of problem), and spirituality.

Further research utilizing the Neuman Systems Model is needed to investigate how families in the United States cope with mental illness. Specifically, research related to the environmental factors that influence a family member's overall coping ability, such as the length of time coping with a mentally ill relative, need to be investigated. Also, further research is needed regarding how specific demographic factors influence the family's ability to cope with mental illness. Identifying variables that influence the family's coping ability may improve the health and functioning of the family.

Using the Neuman Systems Model to help understand the environmental forces that impact the client system will offer nurses insight into the family's coping ability. Nurses must remain sensitive to the stigma associated with mental illness and strive to promote mental health awareness. Advocating mental health services is another role in which nurses are able to affect patient outcomes positively. Also, nursing researchers should continue to investigate the impact that mental illness has on the family. By learning more about how families cope with mental illness, nurses will be able to provide interventions that support healthy family functioning.

Further research utilizing the Neuman Systems Model is needed to investigate how families in the United States cope with mental illness. Specifically, research related to the environmental factors that influence a family member's overall coping ability, such as the length of time coping with a mentally ill relative, need to be investigated. Also, further research is needed regarding how specific demographic factors influence the family's ability to cope with mental illness. Identifying variables that influence the family's coping ability may improve the health and functioning of the family.

## Figures and Tables

**Table 1:** F-COPES subscales per item.

Variable	Mdn	25th Percentile	75th Percentile
Acquiring social support^a^	3.11	2.56	3.67
Reframing	3.75	3.38	4.13
Seeking spiritual support^b^	4.00	3.00	4.50
Mobilizing family to acquire/accept help	3.75	3.00	4.25
Passive appraisal	3.75	3.25	4.50

*Note*. F-COPES [25].

^a^Strongest coping strategy. ^b^Weakest coping strategy.

**Table 2:** Support systems subscales per item.

Variable	Mdn	25th Percentile	75th Percentile
Acquiring social support	3.11	2.56	3.67
Seeking spiritual support	4	3	4.50
Mobilizing family to acquire/accept help	3.75	3	4.25

*Note*. Item analysis reflects median response of coping support systems.

**Table 3:** Demographic and coping correlation.

Variable	*df*	*MS*	*F*	Sig.	Total *R* ^2^
Age	6	164.00	0.57	0.75	0.08
Ethnicity	2	237.19	0.87	0.43	0.04
Gender	1	191.29	0.70	0.41	0.02
Annual income	10	354.11	1.26	0.30	0.33
Relation	5	210.08	0.75	0.59	0.09

*Note*. No correlation significant at *P* < .05, two tailed.
